# Inhibition of Female and Male Human Detrusor Smooth Muscle Contraction by the Rac Inhibitors EHT1864 and NSC23766

**DOI:** 10.3389/fphar.2020.00409

**Published:** 2020-04-07

**Authors:** Bingsheng Li, Qingfeng Yu, Ruixiao Wang, Christian Gratzke, Xiaolong Wang, Annabel Spek, Annika Herlemann, Alexander Tamalunas, Frank Strittmatter, Raphaela Waidelich, Christian G. Stief, Martin Hennenberg

**Affiliations:** ^1^Department of Urology, University Hospital, LMU Munich, Munich, Germany; ^2^Department of Urology, University of Freiburg, Freiburg, Germany

**Keywords:** lower urinary tract symptoms (LUTS), overactive bladder (OAB), detrusor overactivity, bladder smooth muscle contraction, Rac GTPases

## Abstract

**Introduction:**

Lower urinary tract symptoms (LUTS) due to overactive bladder (OAB) are caused by spontaneous detrusor contractions. Medical treatment with muscarinic receptor antagonists or β_3_-adrenoceptor agonists aims to inhibit detrusor contractions, but overall results are unsatisfactory. Consequently, improved understanding of bladder smooth muscle contraction and identification of novel compounds for its inhibition are needed to develop alternative options. A role of the GTPase Rac1 for smooth muscle contraction has been reported from the prostate, but is unknown in the human detrusor. Here, we examined effects of the Rac inhibitors NSC23766, which may also antagonize muscarinic receptors, and EHT1864 on contraction of human detrusor tissues.

**Methods:**

Female and male human detrusor tissues were obtained from radical cystectomy. Effects of NSC23766 (100 µM) and EHT1864 (100 µM) on detrusor contractions were studied in an organ bath.

**Results:**

Electric field stimulation induced frequency-dependent contractions of detrusor tissues, which were inhibited by NSC23766 and EHT1864. Carbachol induced concentration-dependent contractions. Concentration response curves for carbachol were shifted to the right by NSC23766, reflected by increased EC_50_ values, but unchanged E_max_ values. EHT1864 reduced carbachol-induced contractions, resulting in reduced E_max_ values for carbachol. The thromboxane analog U46619 induced concentration-dependent contractions, which remained unchanged by NSC23766, but were reduced by EHT1864.

**Conclusions:**

NSC23766 and EHT1864 inhibit female and male human detrusor contractions. NSC23766, but not EHT1864 competitively antagonizes muscarinic receptors. In addition to neurogenic and cholinergic contractions, EHT1864 inhibits thromboxane A_2_-induced detrusor contractions. The latter may be promising, as the origin of spontaneous detrusor contractions in OAB is noncholinergic. *In vivo*, both compounds may improve OAB-related LUTS.

## Introduction

Storage symptoms affected up to 2.7 billion female and male patients worldwide in 2018, and are commonly caused by overactive bladder (OAB) (Andersson and Arner, 2004; Irwin et al., 2011; Milsom et al., 2014; Powell et al., 2018). Symptoms resulting from OAB include urgency, frequency, nocturia, or incontinence, which are caused by spontaneous, exaggerated contractions of bladder smooth muscle, referred to as detrusor overactivity (DO) (Andersson and Arner, 2004). Consequently, detrusor smooth muscle contraction is the target for medical treatment of storage symptoms and OAB (Oelke et al., 2013; Nambiar et al., 2018). Bladder emptying in a healthy condition is caused by detrusor contractions induced by neurogenic activation of muscarinic acetylcholine receptors on bladder smooth muscle cells (Andersson and Arner, 2004; Andersson, 2011). Although the origin of spontaneous detrusor contractions is not cholinergic and does not result from neurotransmission, muscarinic receptor antagonists (“anticholinergics”) are currently the first line option for medical treatment of storage symptoms (Akino et al., 2008; Oelke et al., 2013; Kushida and Fry, 2015; Nambiar et al., 2018). It has been assumed, that anticholinergics improve symptoms by inhibition of detrusor contractions (Andersson and Arner, 2004; Andersson, 2011).

Despite their prominent role in medical treatment of OAB and storage symptoms, the efficacy of anticholinergics is limited (Oelke et al., 2013; Nambiar et al., 2018). Certainly, reported efficacies vary between studies, as they depend on numerous variables, including study populations or endpoints. In fact, however, up to 45%–65% of patients with storage symptoms are not satisfied by treatment with anticholinergics (Sexton et al., 2011). Due to low efficacy and unbalanced side effects, discontinuation rates are high, amounting up to 90% one year after first prescription (Sexton et al., 2011). At least partially, the limited efficacy of anticholinergics may be explained by noncholinergic detrusor contractions. Spontaneous contractions, which occur in OAB, are supposed to be caused by adenosine triphosphate (ATP) (Akino et al., 2008; Kushida and Fry, 2016; McCarthy et al., 2019). In addition, different prostanoids occur or are even produced within the bladder, which may induce detrusor contraction independently from other mediators or in concert with them (Collins et al., 2009; Rahnama’i et al., 2012). Thus, several prostanoids, including thromboxane A_2_ may cause detrusor smooth muscle contraction directly by activation of corresponding receptors located on smooth muscle cells, but also by prejunctional receptors enhancing the acetylcholine release from cholinergic nerves (Palea et al., 1998; Rahnama’i et al., 2012).

As an alternative option, β_3_-adrenoceptor agonists have been recently introduced for treatment of storage symptoms (Nambiar et al., 2018). They may improve storage symptoms by decreasing the bladder smooth muscle tone by inhibition of cholinergic nerve activity *via* retrograde release of adenosine (D’Agostino et al., 2000; Chapple et al., 2014; Silva et al., 2017; Igawa et al., 2019; Silva et al., 2019). However, it becomes increasingly clear, that their efficacy is not higher than that of anticholinergics (Nambiar et al., 2018), so that the overall situation regarding medical treatment of OAB and storage symptoms still remains inadequate. Considering the limited efficacy of available medications, high discontinuation rates, and the age-dependency of prevalence together with the expected demographic transition, novel options are of high demand (Sexton et al., 2011). Development of such options requires appropriate understanding of bladder smooth muscle contractions, as well as identification of putative new targets and new candidate compounds.

RacGTPases belong to the superfamily of small monomeric GTPases (Takai et al., 2001; Wennerberg and Der, 2004). In addition to their involvement in actin organization and cell cycle progression, a possible Rac-dependent control of smooth muscle contractions has been repeatedly suggested in recent years. Thus, contractions of human prostate tissues can be inhibited by inhibitors for RacGTPases (Wang et al., 2015; Yu et al., 2019). Other studies suggested a role of RacGTPases in smooth muscle contraction of airways, vessels, ileum, and urinary bladder in mice (Rahman et al., 2014; Andre-Gregoire et al., 2017). Consequently, an inhibition of human bladder smooth muscle contraction by Rac inhibitors appears possible, but has to the best of our knowledge not been reported to date. Here, we examined effects of two Rac inhibitors, EHT1864 and NSC23766, on neurogenic, cholinergic, and thromboxane A_2_-induced contractions of female and male human detrusor tissues.

## Materials and Methods

### Human Tissues

Detrusor tissues from 32 female and 38 male patients undergoing radical cystectomy for bladder cancer were collected between 2015 and 2019. This study was carried out in accordance with the Declaration of Helsinki of the World Medical Association, and has been approved by the ethics committee of the Ludwig-Maximilians University, Munich, Germany. Informed consent was obtained from all patients. All samples and data were collected and analyzed anonymously. Accordingly, no patients’ data were collected, stored, or analyzed in the context of this study, and samples were not grouped for pathologic backgrounds or any other condition. Sampling and macroscopic inspection of bladders for tumor burden were performed by pathologists within approximately 30 min following removal of bladders from patients. Organ bath studies were started within 1 h following sampling, i.e., approximately 1.5 h following surgical removal of the organs. For transport and storage, organs and tissues were stored in Custodiol^®^ solution (Köhler, Bensheim, Germany). For macroscopic examination and sampling of detrusor tissues, the bladder was opened by cutting from the bladder outlet to the bladder dome. Subsequently, the intravesical surface and bladder wall were checked macroscopically for tumor infiltration. Tissues were taken from the inner lateral bladder wall, provided that tumor burden in the bladder wall allowed sampling. Urothelial layers were removed from samples.

### Tension Measurements

Strips of detrusor tissues (6 mm × 3 mm × 3 mm) were mounted in 10 ml aerated (95% O_2_ and 5% CO_2_) tissue baths (Danish Myotechnology, Aarhus, Denmark), containing Krebs-Henseleit solution (37°C, pH 7.4) with following composition: 118 mM NaCl, 4.7 mM KCl, 2.55 mM CaCl_2_, 1.2 mM KH_2_PO_4_, 1.2 mM MgSO_4_, 25 mM NaHCO_3_, and 7.5 mM glucose. Preparations were stretched to 4.9 mN and left to equilibrate for 45 min. In the initial phase of the equilibration period, spontaneous decreases in tone are usually observed. Therefore, tension was adjusted three times during the equilibration period, until a stable resting tone of 4.9 mN was attained. After the equilibration period, maximum contraction induced by 80 mM KCl was assessed. Highmolar KCl resulted in biphasic responses, characterized by a phasic contraction reaching a peak within few minutes after addition of KCl (which was used for normalization, as described below), followed by a decline to a tonic, stable contraction (**Supplementary Figure 1**). After the peak of the tonic contraction was obtained and contraction levels started to decline to the tonic phase, chambers were washed three times with Krebs-Henseleit solution for a total of 30 min, and a stable resting tone (close to the first baseline before KCl) was attained (**Supplementary Figure 2**). Thereafter, EHT1864 (final concentration 100 µM), NSC23766 (final concentration 100 µM), or equivalent volumes of dimethylsulfoxide (DMSO) (control for NSC23766) or water (control for EHT1864) were added, and cumulative concentration response curves for carbachol and U46619, or frequency response curves induced by electric field stimulation (EFS) were created 30 min later. Stock solutions of both inhibitors had concentrations of 10 mM, so that 100 µl of stock solutions or of corresponding solvent were added to organ bath chambers. Each chamber contained 10 ml Krebs-Henseleit solution, resulting in concentrations of 0.99% for DMSO and for solvent-related water.

Application of EFS simulates action potentials, resulting in the release of endogenous neurotransmitters, including acetylcholine. For EFS, tissue strips were placed between two parallel platinum electrodes connected to a CS4 stimulator (Danish Myotechnology, Denmark). Square pulses with durations of 1 ms were applied with a voltage of 20 V, for a train duration of 10 s. EFS-induced contractile responses were studied at frequencies of 2, 4, 8, 16, and 32 Hz, with train intervals of 30 s between stimulations.

For calculation of agonist- or EFS-induced contractions, tensions (peak height in EFS-induced contractions and maximum contractions following agonist-exposure) were expressed as % of 80 mM KCl-induced contractions (maximum of phasic contraction). This may correct different smooth muscle or tissue composition, individual variations between bladders of different patients, or any other heterogeneity between different bladders, which may result from different pathologic backgrounds. Only one curve was recorded with each sample (agonist or EFS, either for EHT1864, NSC23766, or corresponding controls). In each single experiment, samples from the same bladder were used for one inhibitor and one control group, so that both curves in each diagram were obtained using the same bladder. E_max_ values, EC_50_ values for contractile agonists, and frequencies (f) inducing 50% of the maximum EFS-induced contraction (Ef_50_) were calculated by curve fitting for each single experiment using GraphPad Prism 6 (Statcon, Witzenhausen, Germany), and analyzed as described below. EC_50_ values have been expressed as –log (M).

### Drugs and Nomenclature

5-(5-(7-(Trifluoromethyl)quinolin-4-ylthio)pentoxyl)-2-(morpholinomethyl)-4H-pyran-4-one dihydrochloride (EHT1864) and N6-[2-[[4-(Diethylamino)-1-methylbutyl]amino]-6-methyl-4-pyrimidinyl]-2-methyl-4,6-quinolinediamine trihydrochloride (NSC23766) are structurally unrelated inhibitors of Rac GTPases (Gao et al., 2004; Akbar et al., 2006; Shutes et al., 2007). Stock solutions (10 mM) were prepared with DMSO (NSC23766) or water (EHT1864), and kept at −20°C until use. Carbachol (carbamoylcholine) is a muscarinic acetylcholine receptor agonist. Aqueous stock solutions of carbachol (10 mM) and dilutions were freshly prepared before each experiment. U46619 ((Z)-7-[(1S,4R,5R,6S)-5-[(E,3S)-3-hydroxyoct-1-enyl]-3-oxabicyclo[2.2.1]heptan-6-yl]hept-5-enoic acid) is an agonist of the thromboxane A_2_ receptor and was dissolved in ethanol. As thromboxane A_2_ has a very short half-life of approximately 32 seconds, U46619 is commonly used as a thromboxane A_2_ receptor agonist (Malmsten, 1976; Shen and Tai, 1998). Stock solutions (10 mM) were stored at −80°C until use. NSC23766, EHT1864, and U46619 were obtained from Tocris (Bristol, UK), and carbachol from Sigma (Munich, Germany).

NSC23766 and EHT1864 were here applied in concentrations of 100 µM. This concentration was used, as it inhibited smooth muscle contractions of human prostate tissues in previous studies (Wang et al., 2015; Yu et al., 2019). This was paralleled by inhibition of Rac1, but not of the closely related GTPase RhoA (Wang et al., 2015). Both inhibitors inhibit RacGTPases but probably by different mechanisms. NSC23766 inhibits RacGTPases by preventing Rac interaction with Rac-specific guanosine nucleotide exchange factors (GEFs), which are upstream activators of GTPases (Gao et al., 2004; Akbar et al., 2006). Its selectivity may be higher for Rac1 than for Rac2 or Rac3, while inhibition of RhoA was not observed (Gao et al., 2004; Akbar et al., 2006). Thus, in biochemical assays and in cultured cells, NSC23766 inhibited Rac1 with an IC_50_ value ranging at 50 µM, whereas even 200 µM did not inhibit RhoA (Gao et al., 2004). EHT1864 binds with high affinity to RacGTPases in biochemical assays *in vitro* (K_D_ = 40 nM for Rac1, 50 nM for Rac1b, 60 nM for Rac2, 250 nM for Rac3) (Shutes et al., 2007). In contrast to the binding affinity examined in biochemical assays, inhibition of Rac activity by EHT1864 was examined in cell culture models. To the best of our knowledge, available data do not include clear IC_50_ values, but suggest that this ranges around 5–10 µM in cultured cells, while even 25 µM did not affect activities of RhoA or Cdc42 GTPase (Desire et al., 2005; Shutes et al., 2007).

### Statistical Analyses

Data are presented as means ± standard deviation (SD) with the indicated number (n) of independent experiments. One-way analysis of variance (ANOVA) was used for comparison of whole concentration response curves, and two-way ANOVA was used for comparison of contractions at single concentrations. For comparison of paired groups in datasets containing two groups (i.e., E_max_ and pEC_50_ values), a paired Student’s t-test was applied. All tests were performed using the SPSS^®^ version 20 (IBM SPSS Statistics, IBM Corporation, Armonk, New York, USA). P values <0.05 were considered significant. Mean differences (MD) with 95% confidence intervals (CI) were calculated using SPSS^®^ version 20. The present study and analyses were designed to be exploratory, but not designed to test a pre-specified statistical null hypothesis. P values of 0.05 or higher are not indicated, so that data sets without p values do not show significant differences. Therefore, p values reported here should be considered as descriptive and not as hypothesis testing. The minimum number of experiments and group sizes in organ bath experiments was pre-planned as n=5/group, as a calculation of descriptive p values was intended. Thus, data were extracted and analyzed, after at least five experiments of a series were performed. Following this analysis, series were discontinued if no effect was seen in concentration response curves, or if descriptive p values <0.05 were obtained in concentration response curves (at single frequencies/agonist concentrations, and/or between whole groups). If these initial results did not reveal p values <0.05, but suggested that an effect could be expected, series were continued and analyzed again. This procedure was possible, as our study was explorative but not designed to test a pre-specified statistical null-hypothesis. In fact, flexible group sizes have been recommended by guidelines for experimental design and analysis in experimental pharmacology, if data are characterized by large variations, what applies here (Curtis et al., 2015; Curtis et al., 2018). Thus, all groups included in the statistical analyses were based on five or more independent experiments and included tissues from five or more patients in each group. Any comparison (e.g., by statistical tests) was confined to paired samples (i.e., tissues from the same bladder), precluding any comparison between different series (e.g., of inhibitor effects in female and male tissues). According to the paired design (allocation of samples from each tissue to the control and inhibitor groups), groups being compared with each other by statistical tests showed identical group sizes. No data or experiments were excluded from analyses. An exception from these settings are some series of E_max_ and pEC_50_ values, as curve fitting was not possible for all single experiments. Thus, curves did not allow automatic curve fitting in one of five single experiments assessing EHT1864 on carbachol-induced contractions in female tissues (resulting in four unpaired values in both groups), and in one of seven single experiments assessing EHT1864 on U46619-induced contractions in female tissues (only in the EHT1864 group), so that calculation of E_max_ and EC_50_ values was not possible for these single experiments. Consequently, statistical analyses were omitted from analyses of these series, as group sizes for these values were below n=5.

## Results

### Effects of NSC23766 on EFS-Induced Detrusor Contractions

**Figure 1:**
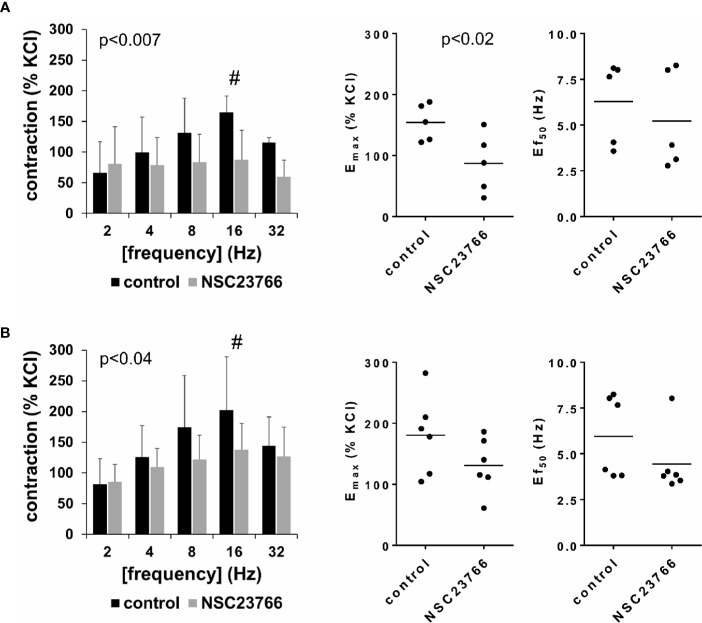
Effects of NSC23766 on electric field stimulation (EFS)–induced contractions of human detrusor tissues. Contractions of female **(A)** and male **(B)** tissues from the lateral urinary bladder wall were induced by EFS in an organ bath, 30 min after addition of NSC23766 (100 µM) or an equivalent amount of dimethylsulfoxide (DMSO) (controls), which was used as solvent for NSC23766. The stock solution of NSC23766 had a concentration of 10 mM, so that 100 µl of stock solution or DMSO was added to organ bath chambers. Each chamber contained 10 ml Krebs-Henseleit solution, resulting in a concentration of 0.99% for DMSO. Shown are data from n=5 female and n=6 male patients, which are means ± SD in frequency response curves ^(#^p < 0.05 for control vs. NSC23766 by two-way ANOVA, and p values for whole groups in inserts from 1-way ANOVA), and E_max_ values and frequencies inducing 50% of the maximum EFS-induced contraction (Ef_50_) for single experiments (calculated by curve fitting) in scatter plots (p value from paired Student’s t-test).

EFS (2–32 Hz) induced frequency-dependent contractions of female and male detrusor tissues, which were reduced by NSC23766 (100 µM) (**Figure 1**, **Supplementary Figure 3**). Following application of NSC23766, EFS-induced contractions were lower than contractions in corresponding controls, i.e., after application of DMSO (p < 0.007 for controls vs. NSC23766 in female tissues, p < 0.04 for controls vs. NSC23766 in male tissues, for whole groups) (**Figure 1**, **Table 1**).

**Table 1:** Mean differences (MD) for EFS-induced contractions after application of NSC23766 (100 µM), EHT1864 (100 µM), or dimethylsulfoxide (DMSO) or water as controls, shown for each frequency and with 95% confidence intervals (CI) (in square brackets, low to high) (% of KCl-induced contractions). Differences in contraction at given agonist concentrations were calculated for each single experiment (i.e., between inhibitor and control group, for corresponding, paired samples from the same bladder in each single experiment, and are expressed as MD with 95% CI. Data are based on experiments on series with n=5 female and n=6 male bladders for NSC23766, and n=5 female and n=5 male bladders for EHT1864.

		EFS, frequency				
		2 Hz	4 Hz	8 Hz	16 Hz	32 Hz
**NSC23766**	**female**	14.4 [−29.8 to 58.6]	−20.6 [−70.9 to 29.6]	−47.7 −98.4 to 3.0]	−76.9 [−130.4 to −23.3]	−55.4 [−93.2 to −17.6]
	**male**	3.6 [−36.6 to 43.8]	−16.7 [−57.1 to 23.7]	−52.8 [−119.5 to 13.9]	−64.8 [−133.2 to 3.6]	−17.1 [−53.1 to 18.9]
**EHT1864**	**female**	−59.0 [−112.0 to −6.0]	−72.7 [−111.7 to −33.8]	−88.3 [−135.3 to −41.3]	−131.1 [−197 to −64.7]	−128.3 [−174 to −82.4]
	**male**	−35.2 [−65.4 to −5.0]	−83.2 [−185.9 to 19.6]	−103.2 [−241.6 to 35.2]	−129.9 [−281.2 to 21.5]	−90.0 [−169.2 to −10.8]

Inhibition of EFS-induced contractions was confirmed by calculation of E_max_ values by curve fitting, which was possible for all single experiments and all groups. For female tissues, curve fitting revealed E_max_ values for EFS-induced contractions of 155% ± 30% of KCl-induced contractions following application of DMSO, and 87% ± 49% following application of NSC23766 (p < 0.02) (MD −67% of KCl-induced contraction [95% CI: −111 to −23]) (**Figure 1A**). For male tissues, curve fitting revealed E_max_ values for EFS-induced contractions of 181% ± 65% of KCl-induced contractions following application of DMSO, and 131% ± 45% following application of NSC23766 (p=0.057) (MD −50% of KCl-induced contraction [95% CI: −101 to 2.1]) (**Figure 1B**).

Calculation of frequencies inducing 50% of the maximum EFS-induced contraction (Ef_50_) by curve fitting was possible for all single experiments and all groups, and suggested no effect of NSC23766 on Ef_50_ values. For EFS-induced contractions of female tissues, curve fitting revealed Ef_50_ values of 6.3 Hz ±2.3 Hz following application of DMSO, and 5.2 Hz ±2.7 Hz following application of NSC23766 (MD −1.1 Hz [95% CI: −5.5 to 3.4]) (**Figure 1A**). For EFS-induced contractions of male tissues, curve fitting revealed Ef_50_ values of 6.0 ± 2.2 Hz following application of DMSO, and 4.4 Hz ±1.7 Hz following application of NSC23766 (MD −1.5 Hz [95% CI: −4.0 to 1.0]) (**Figure 1B**).

### Effects of EHT1864 on EFS-Induced Detrusor Contractions

**Figure 2:**
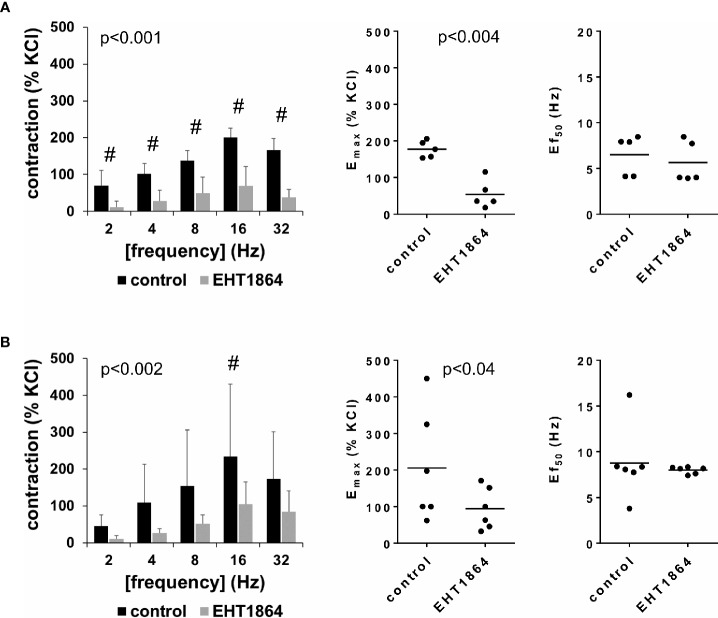
Effects of EHT1864 on electric field stimulation (EFS)–induced contractions of human detrusor tissues. Contractions of female **(A)** and male **(B)** tissues from the lateral urinary bladder wall were induced by EFS in an organ bath, 30 min after addition of EHT1864 (100 µM) or an equivalent amount of water (controls), which was used as solvent for EHT1864. The stock solution of EHT1864 had a concentration of 10 mM, so that 100 µl of water was added to organ bath chambers. Each chamber contained 10 ml Krebs-Henseleit solution, resulting in a concentration of 0.99% for solvent-related water. Shown are data from n=5 female and n=6 male patients, which are means ± SD in frequency response curves ^(#^p < 0.05 for control vs. EHT1864 by two-way ANOVA, and p values for whole groups in inserts from 1-way ANOVA), and E_max_ values and frequencies inducing 50% of the maximum EFS-induced contraction (Ef_50_) for single experiments (calculated by curve fitting) in scatter plots (p value from paired Student’s t-test).

EFS-induced contractions of female and male detrusor tissues were reduced by EHT1864 (100 µM) (**Figure 2**, **Supplementary Figure 3**). Following application of EHT1864, EFS-induced contractions were lower than contractions in corresponding controls, i.e., after application of water (p < 0.001 for controls vs. EHT1864 in female tissues, p < 0.002 for controls vs. EHT1864 in male tissues, for whole groups) (**Figure 2**, **Table 1**).

Inhibition of EFS-induced contractions was confirmed by calculation of E_max_ values by curve fitting, which was possible for all single experiments and all groups. For female tissues, curve fitting revealed E_max_ values for EFS-induced contractions of 178% ± 23% of KCl-induced contractions following application of water, and 54% ± 39% following application of EHT1864 (p < 0.004) (MD −124% of KCl-induced contraction [95% CI: −179 to −69]) (**Figure 2A**). For male tissues, curve fitting revealed E_max_ values for EFS-induced contractions of 206% ± 153% of KCl-induced contractions following application of water, and 94% ± 57% following application of EHT1864 (p=0.04) (MD −112% of KCl-induced contraction [95% CI: −215 to −8]) (**Figure 2B**).

Calculation of Ef_50_ values by curve fitting was possible for all single experiments and all groups, and suggested no effect of EHT1864 on Ef_50_ values for frequencies in EFS. For EFS-induced contractions of female tissues, curve fitting revealed Ef_50_ values of 6.5 Hz ±2.2 Hz following application of water, and 5.6 Hz ±2.3 Hz following application of EHT1864 (MD −0.9 Hz [95% CI: −3.0 to 1.2]) (**Figure 2A**). For EFS-induced contractions of male tissues, curve fitting revealed Ef_50_ values of 8.8 Hz ±4.0 Hz following application of water, and 8.0 ± 0.4 Hz following application of EHT1864 (MD −0.8 Hz [95% CI: −5.0 to 3.4]) (**Figure 2B**).

### Effects of NSC23766 on Carbachol-Induced Detrusor Contractions

**Figure 3:**
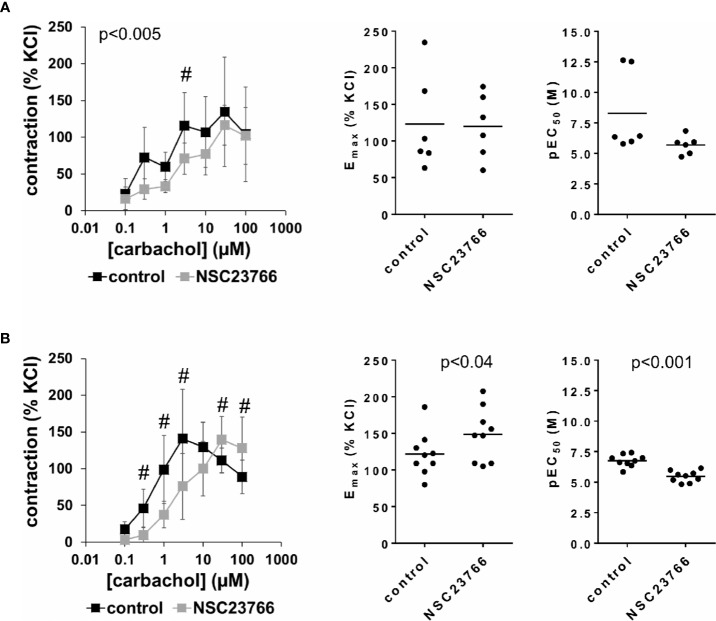
Effects of NSC23766 on carbachol-induced contractions of human detrusor tissues. Contractions of female **(A)** and male **(B)** tissues from the lateral urinary bladder wall were induced by cumulative concentrations of carbachol in an organ bath, 30 min after addition of NSC23766 (100 µM) or an equivalent amount of dimethylsulfoxide (DMSO) (controls), which was used as solvent for NSC23766. The stock solution of NSC23766 had a concentration of 10 mM, so that 100 µl of stock solution or DMSO was added to organ bath chambers. Each chamber contained 10 ml Krebs-Henseleit solution, resulting in a concentration of 0.99% for DMSO. Shown are data from n=6 female and n=9 male patients, which are means ± SD in concentration response curves ^(#^p < 0.05 for control vs. NSC23766 by two-way ANOVA, and p values for whole groups in inserts from 1-way ANOVA), and E_max_ and pEC_50_ values for single experiments (calculated by curve fitting) in scatter plots (p value from paired Student’s t-test).

Carbachol (0.1–100 µM) induced concentration-dependent contractions of female and male detrusor tissues (**Figure 3**). Application of NSC23766 (100 µM) resulted in a rightward shift of concentration response curves for carbachol, which was clearly seen using tissues from male patients, and partially using tissues from female patients (**Figure 3**, **Supplementary Figure 4**). Thus, following application of NSC23766, contractions induced by carbachol concentrations up to 3 µM were lower than contractions in corresponding controls, i.e., after application of DMSO, while maximum contractile forces (observed at carbachol concentrations of 3–30 µM) were not changed (**Figure 3**, **Table 2**). Thus, maximum contractions recovered at high carbachol concentrations (30–100 µM) in the NSC23766 group in both genders (**Figure 3**, **Table 2**). In female bladders, contractions stayed lower or were similar after application of NSC23766 compared to DMSO at all carbachol concentrations (p < 0.005) (**Figure 3A**, **Table 2**). In male bladders, contractions at 30–100 µM carbachol were even higher in the NSC23766 than in the DMSO group (**Figure 3B**, **Table 2**). Consequently, statistical tests to compare the whole groups with each other (control vs. NSC23766) were not meaningful for male bladders.

**Table 2:** Mean differences (MD) for carbachol-induced contractions after application of NSC23766 (100 µM), EHT1864 (100 µM), or DMSO or water as controls, shown for each applied carbachol concentration and with 95% confidence intervals (CI) (in square brackets, low to high) (% of KCl-induced contractions). Differences in contraction at given agonist concentrations were calculated for each single experiment (i.e., between inhibitor and control group, for corresponding, paired samples from the same bladder in each single experiment, and are expressed as MD with 95% CI. Data are based on experiments on series with n=6 female and n=9 male bladders for NSC23766, and n=3 female (see text) and n=5 male bladders for EHT1864 (experiments, where curve fitting was not possible and resulted in unpaired values, were excluded, what concerns two experiments with female bladder and EHT1864).

		carbachol concentration						
		0.1 µM	0.3 µM	1 µM	3 µM	10 µM	30 µM	100 µM
**NSC23766**	**female**	−6.6 [−24.9 to 11.7]	−43.3 [−74.6 to −12.0]	−26.3 [−51.0 to −1.7]	−45.0 [−107.4 to 17.5]	−30.1 [−85.7 to 25.4]	−18.1 [−81.9 to 45.6]	−2.4 [−45.1 to 40.3]
	**male**	−14.1 [−21.5 to −6.7]	−36.5 [−50.6 to −22.5]	−61.3 [−87.1 to −35.5]	−64.8 [−92.4 to −37.2]	−29.4 [−49.5 to −9.4]	28.4 [1.6 to 55.2]	39.1 [10.7 to 67.5]
**EHT1864**	**female**	−51.4 [−92.4 to 10.3]	−53.9 [−70.8 to −36.9]	−51.9 [−90.0 to −13.8]	−31.5 [−63.6 to 0.6]	−52.9 [−102.0 to −3.7]	−52.2 [−101.9 to −2.5]	−53.9 [−111.8 to 4.0]
	**male**	−30.2 [−64.3 to 3.8]	−55.3 [−101.5 to −9.0]	−63.3 [−81.9 to −44.6]	−61.5 [−95.7 to −27.3]	−62.6 [−76.7 to −48.6]	−51.1 [−81.7 to −20.6]	−58.0 [−83.6 to −32.4]

The rightward shift of concentration response curves for carbachol was confirmed by calculation of E_max_ and pEC_50_ values by curve fitting, which was possible for all single experiments and all groups. For female tissues, curve fitting revealed E_max_ values for carbachol-induced contractions of 123% ± 65% of KCl-induced contractions following application of DMSO, and 120% ± 44% following application of NSC23766 (p=0.9) (MD −3% of KCl-induced contraction [95% CI: −47 to 40]) (**Figure 3A**). For male tissues, curve fitting revealed E_max_ values for carbachol-induced contractions of 122% ± 30% of KCl-induced contractions following application of DMSO, and 149% ± 36% following application of NSC23766 (p < 0.04) (MD 27% of KCl-induced contraction [95% CI: 3 to 50]) (**Figure 3B**).

Calculation of EC_50_ values by curve fitting was possible for all single experiments and all groups, and reflected the rightward shift of concentration response curves for carbachol by NSC23766 (**Figure 3**). Thus, EC_50_ values for carbachol were increased by NSC23766 in female and male detrusor tissues (**Figure 3**). For female tissues, curve fitting revealed pEC_50_ values of 8.3 ± 3.3 M following application of DMSO, and 5.7 ± 0.8 M following application of NSC23766 (p=0.07) (MD −2.6 [95% CI: −5.6 to 0.4]) (**Figure 3A**). For male tissues, curve fitting revealed pEC_50_ values of 6.8 ± 0.5 M following application of DMSO, and 5.5 ± 0.5 M following application of EHT1864 (p < 0.001) (MD −1.3 [95% CI: −1.6 to −1.0]) (**Figure 3B**).

### Effects of EHT1864 on Carbachol-Induced Detrusor Contractions

**Figure 4:**
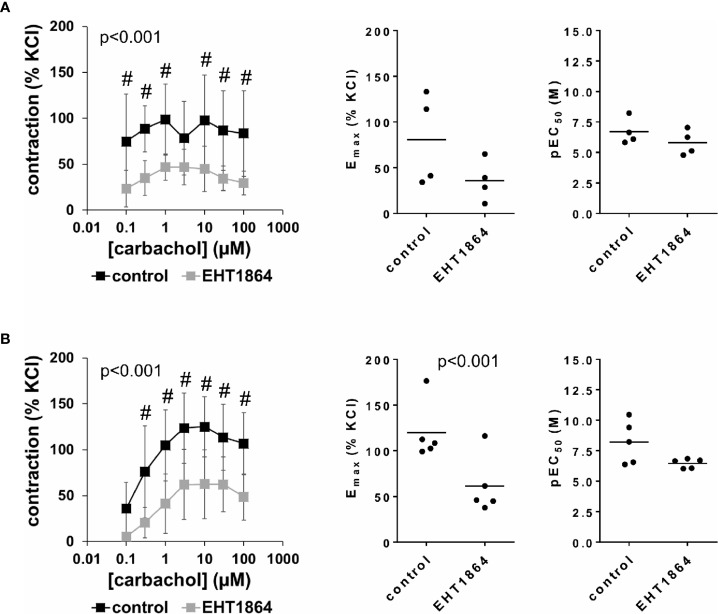
Effects of EHT1864 on carbachol-induced contractions of human detrusor tissues. Contractions of female **(A)** and male **(B)** tissues from the lateral urinary bladder wall were induced by cumulative concentrations of carbachol in an organ bath, 30 min after addition of EHT1864 (100 µM) or an equivalent amount of water (controls), which was used as solvent for EHT1864. The stock solution of EHT1864 had a concentration of 10 mM, so that 100 µl of water was added to organ bath chambers. Each chamber contained 10 ml Krebs-Henseleit solution, resulting in a concentration of 0.99% for solvent-related water. Shown are data from n=5 female and n=5 male patients, which are means ± SD in concentration response curves (^#^p < 0.05 for control vs. EHT1864 by two-way ANOVA, and p values for whole groups in inserts from 1-way ANOVA), and E_max_ and pEC_50_ values for single experiments (calculated by curve fitting) where calculation was possible (see text) in scatter plots (p value from paired Student’s t-test).

Carbachol-induced contractions of female and male detrusor tissues were reduced by EHT1864 (**Figure 4**, **Supplementary Figure 4**). Following application of EHT1864, carbachol-induced contractions were lower than contractions in corresponding controls, i.e., after application of water (p < 0.001 for controls vs. EHT1864 in female and male tissues, for whole groups) (**Figure 4**, **Table 2**). A rightward shift, as it was seen for NSC23766 in carbachol-induced contraction at least for male tissues, was not observed for EHT1864 (**Figure 4**).

Inhibition of carbachol-induced contractions was confirmed by calculation of E_max_ values by curve fitting. For female tissues, curve fitting was possible for four of five single experiments, in both the control and the EHT1864 group, so that statistical analysis was omitted for these values. Curve fitting revealed E_max_ values for carbachol-induced contractions of female detrusor of 81% ± 50% of KCl-induced contractions following application of water, and 36% ± 23% following application of EHT1864 (MD −37% of KCl-induced contraction [95% CI: −125 to 51], on the basis of three available, paired values) (**Figure 4A**). For male tissues, curve fitting was possible for all single experiments and both groups, and revealed E_max_ values for carbachol-induced contractions of 120% ± 32% of KCl-induced contractions following application of water, and 61% ± 32% following application of EHT1864 (p < 0.001) (MD −59% of KCl-induced contraction [95% CI: −74 to −43]) (**Figure 4B**).

Calculation of EC_50_ values for female detrusor tissues by curve fitting was possible for four of five single experiments, in both the control and the EHT1864 group, and for all single experiments of this series for male tissues. A small increase of EC_50_ values was observed for male bladders, while no effect was seen for female bladders (**Figure 4**). Thus, curve fitting revealed pEC_50_ values of 6.7 M ±1.1 M following application of water and 5.8 M ±1.0 M following application of EHT1864 for carbachol in female tissues (**Figure 4A**) (MD −0.5 [95% CI: −4.0 to 3.0], on the basis of three available, paired values), and 8.2 M ±1.8 M following application of water and 6.5 M ±0.4 M following application of EHT1864 (p=0.1) (MD −1.8 [95% CI: −3.9 to 0.4]) for carbachol in male tissues (**Figure 4B**).

### Effects of NSC23766 on U46619-Induced Detrusor Contractions

**Figure 5:**
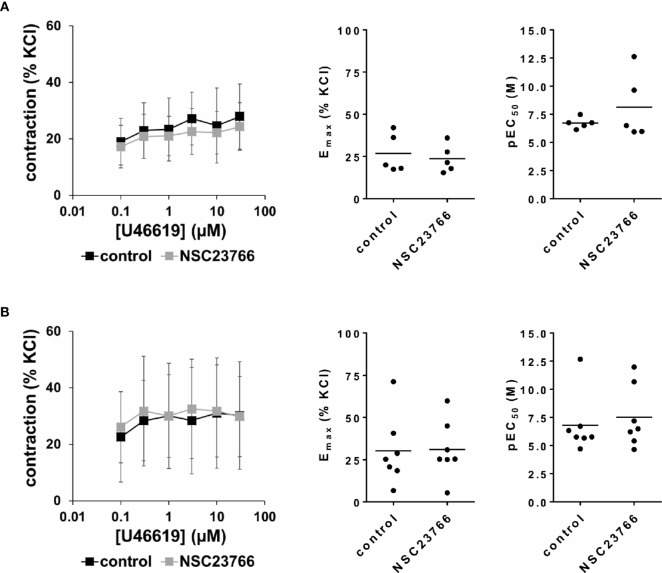
Effects of NSC23766 on U46619-induced contractions of human detrusor tissues. Contractions of female **(A)** and male **(B)** tissues from the lateral urinary bladder wall were induced by cumulative concentrations of the thromboxane A_2_ analog U46619 in an organ bath, 30 min after addition of NSC23766 (100 µM) or an equivalent amount of dimethylsulfoxide (DMSO) (controls), which was used as solvent for NSC23766. The stock solution of NSC23766 had a concentration of 10 mM, so that 100 µl of stock solution or DMSO were added to organ bath chambers. Each chamber contained 10 ml Krebs-Henseleit solution, resulting in a concentration of 0.99% for DMSO. Shown are data from n=5 female and n=7 male patients, which are means ± SD in concentration response curves, and E_max_ and pEC_50_ values for single experiments (calculated by curve fitting) in scatter plots (p value from paired Student’s t-test).

U46619 (0.1–30 µM) induced concentration-dependent contractions of female and male detrusor tissues (**Figure 5**). Application of NSC23766 (100 µM) did not change concentration response curves for U46619 (**Figure 5**, **Table 3**).

**Table 3:** Mean differences (MD) for U46619-induced contractions after application of NSC23766 (100 µM), EHT1864 (100 µM), or dimethylsulfoxide (DMSO) or water as controls, shown for each applied U46619 concentration and with 95% confidence intervals (CI) (in square brackets, low to high) (% of KCl-induced contractions). Differences in contraction at given agonist concentrations were calculated for each single experiment (i.e., between inhibitor and control group, for corresponding, paired samples from the same bladder in each single experiment, and are expressed as MD with 95% CI. Data are based on experiments on series with n=5 female and n=7 male bladders for NSC23766, and n=5 female and n=5 male bladders for EHT1864 (experiments, where curve fitting was not possible and resulted in unpaired values, were excluded, what concerns one experiment with female bladder and EHT1864).

		U46619 concentration					
		0.1 µM	0.3 µM	1 µM	3 µM	10 µM	**30 µM**
**NSC23766**	**female**	−1.7 [−11.9 to 8.4]	−2.1 [−13.2 to 9.1]	−2.3 [−14.6 to 10.0]	−4.6 [−16.7 to 7.5]	−2.5 [−15.8 to 10.7]	−3.6 [−16.0 to 8.7]
	**male**	3.5 [−9.8 to 16.8]	3.4 [−10.1 to 16.8]	0.0 [−11.8 to 11.7]	4.1 [−5.4 to 13.7]	0.7 [−11.1 to 12.6]	−0.4 [−12.8 to 12.0]
**EHT1864**	**female**	−7.4 [−13.9 to −0.9]	−8.4 [−19.0 to 2.2]	−10.1 [−19.3 to −0.9]	−10.4 [−21.7 to 1.0]	−9.2 [−23.5 to 5.1]	−11.2 [−30.6 to 8.1]
	**male**	−7.2 [−20.4 to 6.0]	−8.6 [−23.9 to 6.8]	−9.7 [−25.3 to 5.9]	−7.8 [−20.8 to 5.1]	−6.5 [−19.1 to 6.1]	−8.6 [−16.9 to −0.3]

The effect of NSC23766 on concentration response curves for U46619 was confirmed by calculation of E_max_ values by curve fitting, which was possible for all single experiments and all groups in both genders. For female tissues, curve fitting revealed E_max_ values for U46619-induced contractions of 27% ± 12% of KCl-induced contractions following application of DMSO, and 24% ± 8% following application of NSC23766 (p=0.6) (MD −3% of KCl-induced contraction [95% CI: −16 to 10]) (**Figure 5A**). For male tissues, curve fitting revealed E_max_ values for U46619-induced contractions of 30% ± 21% of KCl-induced contractions following application of DMSO, and 31% ± 17% following application of NSC23766 (p=0.9) (MD 1% of KCl-induced contraction [95% CI: −12 to 13]) (**Figure 5B**).

Calculation of EC_50_ values by curve fitting was possible for all single experiments and all groups in both genders. Although no curve shift was observed in concentration response curves, calculation of pEC_50_ values suggested decreases of EC_50_ values for U46619 (**Figure 5**). However, this may concern only two from five experiments in female, and two from seven experiments in male tissues (**Figure 5**). Thus, curve fitting revealed pEC_50_ values of 6.7 ± 0.5 M following application of DMSO and 8.1 ± 3 M following application of NSC23766 (p=0.3) for U46619 in female tissues (MD 1.4 [95% CI: −2.8 to 5.6]) (**Figure 5A**), and 6.8 ± 2.7 M following application of DMSO and 7.5 ± 2.7 M following application of NSC23766 (p=0.6) (MD 0.7 [95% CI: −1.4 to 2.8]) for U46619 in male tissues (**Figure 5B**).

### Effects of EHT1864 on U46619-Induced Detrusor Contractions

**Figure 6:**
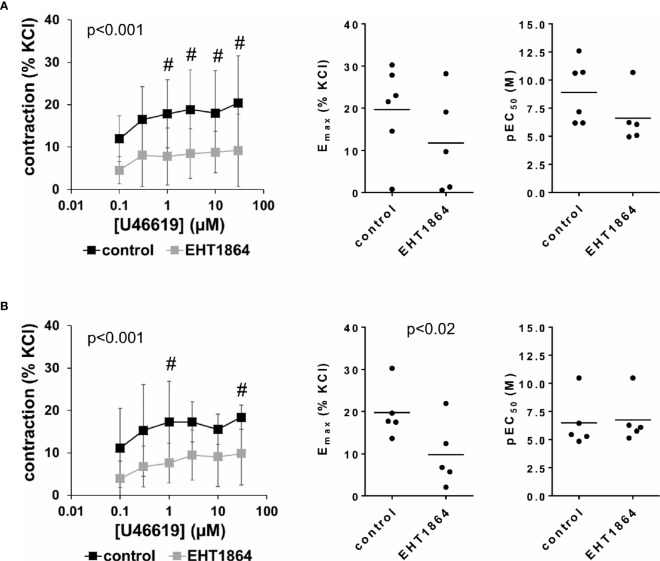
Effects of EHT1864 on U46619-induced contractions of human detrusor tissues. Contractions of female **(A)** and male **(B)** tissues from the lateral urinary bladder wall were induced by cumulative concentrations of the thromboxane A_2_ analog U46619 in an organ bath, 30 min after addition of EHT1864 (100 µM) or an equivalent amount of water (controls), which was used as solvent for EHT1864. The stock solution of EHT1864 had a concentration of 10 mM, so that 100 µl of water was added to organ bath chambers. Each chamber contained 10 ml Krebs-Henseleit solution, resulting in a concentration of 0.99% for solvent-related water. Shown are data from n=6 female and n=5 male patients, which are means ± SD in concentration response curves (^#^p < 0.05 for control vs. EHT1864 by two-way ANOVA, and p values for whole groups in inserts from 1-way ANOVA), and E_max_ and pEC_50_ values for single experiments (calculated by curve fitting) where calculation was possible (see text) in scatter plots (p value from paired Student’s t-test).

U46619-induced contractions of female and male detrusor tissues were reduced by EHT1864 (**Figure 6**). Following application of EHT1864, U46619-induced contractions were lower than contractions in corresponding controls, i.e., after application of water (p < 0.001 for controls vs. EHT1864 in female and male tissues, for whole groups) (**Figure 6**, **Table 3**).

Inhibition of U46619-induced contractions was confirmed by calculation of E_max_ values by curve fitting. For female tissues, curve fitting was possible for five of six single experiments in the EHT1864 group and all single experiments in the control group. Due to the unpaired character of these data, statistical analysis was omitted for these values. This revealed E_max_ values for U46619 of 20% ± 11% of KCl-induced contractions following application of water, and 10% ± 12% following application of EHT1864 (MD −9% of KCl-induced contraction [95% CI: −36 to 18], on the basis of five available, paired values) (**Figure 6A**). For male tissues, curve fitting was possible for all single experiments and both groups, and revealed E_max_ values for U46619 of 20% ± 6% of KCl-induced contractions following application of water, and 10% ± 8% following application of EHT1864 (p < 0.02) (MD −10% of KCl-induced contraction [95% CI: −17 to −3]) (**Figure 6B**).

Calculation of EC_50_ values for female detrusor tissues by curve fitting was possible for five of six single experiments in the EHT1864 group and all single experiments in the control group, and revealed pEC_50_ values for U46619 of 8.9 M ±2.7 M following application of water, and 6.6% ± 2.3% following application of EHT1864 (MD −3.1 [95% CI: −8 to 1.9], on the basis of five available, paired values) (**Figure 6A**). For male tissues, curve fitting was possible for all single experiments and both groups, and revealed pEC_50_ values for U46619 of 6.5 M ±2.3 M following application of water, and 6.8 M ±2.1 M following application of EHT1864 (MD 0.2 [95% CI: −0.5 to 1.0]) (**Figure 6B**).

## Discussion

The findings of this study suggest that NSC23766 and EHT1864, two different Rac inhibitors, inhibit smooth muscle contractions of tissues from the female and male human bladder wall. Both inhibitors inhibited EFS-induced contractions in tissues from both genders, which was observed at single frequencies and for calculated E_max_ values. NSC23766 shifted concentration response curves for carbachol to the right in tissues from male patients, and increased the EC_50_ but did not reduce the E_max_ values for carbachol in tissues from both genders. In contrast, EHT1864 reduced the maximum effects of carbachol, which was significant for male tissues, and by trend observed for female tissues. U46619-induced contractions were reduced by EHT1864 in both genders, but remained unaffected by NSC23766.

Rac GTPases occur in three isoforms designated to as Rac1-3, and belong to the superfamily of small monomeric GTPases comprising at least 25 members in addition to Rac, including RhoA (Takai et al., 2001; Wennerberg and Der, 2004). While the role of RhoA for promotion of smooth muscle contraction is well established for all smooth muscle tissues (Somlyo and Somlyo, 2000; Somlyo and Somlyo, 2003; Christ and Andersson, 2007; Puetz et al., 2009), a similar role for RacGTPases has been proposed more recently. NSC23766 and EHT1864 inhibited contractions of human prostate tissues, so that a procontractile role of Rac1 has been proposed for prostate smooth muscle (Wang et al., 2015; Yu et al., 2019). These previous and our present findings for the detrusor and prostate are in line with other studies, where Rac inhibitors inhibited cholinergic, serotonin-, and endothelin-induced contractions of airway tissues from humans, mice and rats, as well as adrenergic and serotonin-induced contractions of rodent vessels, and contractions of the mouse ileum (Staiculescu et al., 2013; Rahman et al., 2014; Shibata et al., 2015; Andre-Gregoire et al., 2017; Harvey et al., 2017; Sakai et al., 2018). This role of Rac suggested by inhibitor experiments has been confirmed by knockout of Rac1 expression in mice, which reduced contractions in airway, vascular and gastrointestinal smooth muscle (Rahman et al., 2014; Andre-Gregoire et al., 2017). Finally, carbachol-induced bladder contractions of mice were inhibited by NSC23766, EHT1864, and Rac1 knockout (Rahman et al., 2014). Here we show, that both inhibitors also inhibit bladder smooth muscle contractions in female and male humans.

We observed that cholinergic detrusor contractions were inhibited by competitive receptor antagonism by NSC23766, and noncompetitively by EHT1864. EHT1864, but not NSC23766 inhibited U46619-induced contractions. Together, this points to divergent pharmacologic profiles of both inhibitors, what is in line with previous findings. NSC23766 acts in fact as a competitive antagonist at muscarinic acetylcholine receptors (Levay et al., 2013), in addition to Rac inhibition. Here, NSC23766 caused rightward shifts for carbachol-induced contractions of male detrusor tissues, resulting in reduced pEC_50_ values, and unchanged maximum contractions in concentration response curves or even elevated calculated E_max_ values, together reflecting antagonism of cholinergic receptors. A typical rightward shift for carbachol-induced contractions did not occur for female tissues. However, conclusions may be similar, as at least a nonsignificant decrease in pEC_50_ values and reduced contractions at low carbachol concentrations, and no decrease of maximum contractions or of calculated E_max_ values were observed. Therefore, and because NSC23766 did not inhibit U46619-induced detrusor contractions, it may be assumed, that muscarinic antagonism may be the predominant mechanism of contraction inhibition by NSC23766 in our study, although an involvement of GEF-mediated Rac activation can not be excluded. Whether gender-specific differences account for the slight differences of NSC23766 effects in female and male tissues or not, e.g., differential expression patterns of Rac isoforms, can not be estimated on the basis of our data.

For the prostate, cytotoxic effects of NSC23766 and EHT1864 were recently excluded to account for contraction inhibition in organ bath experiments, as these effects did not occur quickly enough (Yu et al., 2019). As very similar conditions were applied here to bladder tissues, it appears unlikely, that cytotoxic effects underly the inhibitory effect on detrusor contraction in our study. Details of the possible procontractile role of Rac in detrusor smooth muscle contraction can not be derived on the basis of our present data, and need to be addressed by future studies. Previous studies pointed to organ-specific mechanisms, which may involve myosin light chain phosphorylation and its upstream pathways in vessels and airways, and to actin organization in the prostate (Wang et al., 2015; Shibata et al., 2015; Andre-Gregoire et al., 2017; Sakai et al., 2018). Molecular details explaining the inhibition of smooth muscle contraction observed here using detrusor tissues need to be examined in future studies, and may diverge for both inhibitors, as EHT1864, but not NSC23766 inhibited U46619-induced contractions. Nevertheless, and in line with previous data, our findings suggest a role of Rac in intracellular signaling, which positively regulates detrusor smooth muscle contractions. Previous studies addressing procontractile signaling in bladder smooth muscle focussed on RhoA/Rho kinase, phospholipase C/calcium, and protein kinase C, which are shared by all smooth muscle types (Somlyo and Somlyo, 2000; Somlyo and Somlyo, 2003). With Rac, our present findings may uncover a novel player in human detrusor smooth muscle contraction. This may reflect, that the molecular mechanisms of detrusor smooth muscle contraction are still incompletely understood. Gender differences were principally confined to the above-mentioned differences in effects of NSC23766 on carbachol-induced contractions, pointing to a similar relevance of the NSC23766- and EHT1864-sensitive mechanisms in female and male bladders.

Our study may contain several limitations. The origin of EFS-induced contractions in our tissues has not been characterized. In previous studies, EFS-induced contractions of human detrusor tissues were largely or even completely mediated by neurotransmission (Kinder and Mundy, 1985; Saito et al., 1993; Tonini et al., 1994; Tagliani et al., 1997). It appears likely that this may also apply to our experiments. However, conditions may vary between studies, and in fact, the neurogenic character of these contractions was not confirmed in our study. Similarly, the involved neurotransmitters accounting for EFS-induced contractions in our tisses were not identified. Neurogenic contractions of detrusor tissues are assumed to be largely or solely cholinergic under normal conditions, while purinergic contractions may contribute to detrusor smooth muscle tone under pathologic conditions, including DO and OAB (Saito et al., 1993; Palea et al., 1995; D’Agostino et al., 2000; Elliott et al., 2013; Andersson, 2015; Kushida and Fry, 2015; Andersson et al., 2018). The identity of neurotransmitters, a possible purinergic contribution to smooth muscle tone, and the pathological background have not been addressed in our tissues. Tissues for our study were obtained from patients undergoing radical cystectomy for bladder cancer, and were not grouped for OAB or LUTS, but anonymized after sampling. Accordingly, no patients’ data were stored or analyzed in the context of our study. Consequently, our findings may not be specific for OAB or DO, and tissues may be characterized by high heterogeneity including different tumor burden, varying degree of storage symptoms, inflammation, or others. A comparison to nondiseased controls was not possible, as such samples are not available under comparable conditions. Together, these heterogeneities may contribute to large variations of EC_50_ values and standard deviations. As force development may depend on mentioned heterogeneities, but also on individual tissue composition, smooth muscle condition, or similar variables, contractions were referred to nerve- and receptor-independent contractions induced by highmolar KCl in our study. For possible individual variations, we only compared differences between paired samples, i.e., between samples obtained from the same bladders. This precluded comparisons between different series, e.g., effects of NSC23766 on EFS between female and male bladders, which appeared slightly different. Notably, it was possible to observe effects of Rac inhibitors despite these limitations, what may underline the relevance of their targets for regulation of detrusor smooth muscle contraction.

Inhibition of detrusor smooth muscle contraction is assumed to underly the beneficial effects of anticholinergics and β_3_-agonists, which are applied for medical treatment of storage symptoms (Andersson and Arner, 2004; Andersson, 2011; Oelke et al., 2013; Nambiar et al., 2018). However, their efficacy is clearly limited (Nambiar et al., 2018). Nevertheless, improvements by anticholinergics are observed, although muscarinic receptors or neurogenic mediators are not responsible for the spontaneous detrusor contractions in OAB (Akino et al., 2008; Kushida and Fry, 2015). It could be speculated that EHT1864 improves storage symptoms with higher efficacy than anticholinergics, as EHT1864 did not only inhibit cholinergic and neurogenic detrusor contractions, but also U46619-induced contractions. Thromboxane A_2_ may act as a paracrine mediator of smooth muscle contraction in the urinary bladder and other organs, including the prostate (Strittmatter et al., 2011; Rahnama’i et al., 2012; Hennenberg et al., 2013; Hennenberg et al., 2017). In OAB, thromboxane A_2_ and other prostanoinds may account for spontaneous detrusor contractions (Collins et al., 2009; Rahnama’i et al., 2012).

Previous animal studies, performed in nonurologic context, demonstrated that NSC23766 and EHT1864 can be applied *in vivo*, where they may be tolerated at least for short time intervals. In addition to inhibition of detrusor contraction shown here, NSC23766 and EHT1864 inhibited prostate smooth muscle contraction and growth of prostate cells, which contribute to bladder outlet obstruction and finally LUTS suggestive of benign prostatic hyperplasia (BPH) (Wang et al., 2015; Yu et al., 2019). Consequently, it appears possible, that Rac inhibitors may interfere with obstructive and storage symptoms at once. In fact, it is now clear that many male patients suffer for both kinds of LUTS, referred to as mixed LUTS (Fullhase et al., 2013; Oelke et al., 2013). To date, combination therapies are still required, which are, however, afflicted by high discontinuation rates (Fullhase et al., 2013). Compounds such as Rac inhibitors may offer the possibility to develop single compound medications for treatment of mixed LUTS. Whether the translational value of NSC23766 and EHT1864 is limited by side effects or not, still needs to be addressed. Hypotensive side effects, similar to those resulting from application of α_1_-adrenoceptor antagonists in male LUTS (Oelke et al., 2013), must not necessarily result from Rac inhibitors. Rather, hypertensive effects could be expected, as Rac1 knockout in mice was associated with hypertension, due to increased vascular resistance resulting from disruption of nitric oxide-induced vasorelaxation (Andre et al., 2014). Notably, the present together with previous findings demonstrate that compounds addressing smooth muscle contraction in the detrusor and prostate, plus prostate growth all at once could be available.

## Conclusions

NSC23766 and EHT1864 inhibit female and male human detrusor contractions, but show divergent pharmacologic profiles. The previously presumed antagonism of NSC23766 at muscarinic receptors is reflected by its effect on neurogenic and cholinergic detrusor contractions. In addition to neurogenic and cholinergic contractions, EHT1864 inhibits thromboxane A_2_-induced detrusor contractions. The latter may be promising, as the origin of spontaneous detrusor contractions in OAB is noncholinergic. *In vivo*, both compounds may improve LUTS attributed to OAB.

## Data Availability Statement

All datasets generated for this study are included in the article/**Supplementary Material**.

## Ethics Statement

The studies involving human participants were reviewed and approved by Ethikkommission bei der LMU München, LMU Munich. The patients/participants provided their written informed consent to participate in this study.

## Author Contributions

CG, CS, and MH contributed conception and design of the study. All authors contributed to collection of human bladder tissues. BL, QY, RW, XW, and AS performed organ bath experiments. BL, QY, RW, and MH analyzed the data. MH wrote the first draft of the manuscript. BL wrote sections of the manuscript. XW, AS, AH, AT, FS, RW, and CG critically revised the manuscript. All authors contributed to manuscript revision, read and approved the submitted version. BL and QY contributed equally.

## Funding

This work was supported by grants from the Deutsche Forschungsgemeinschaft (grants HE 5825/6-1, and GR 3333/6-1), and the Chinese Scholarship Council (CSC), and the Friedrich-Baur-Stiftung (grant 71/16), which were not involved in study design, in collection, analysis and interpretation of data, in writing of the manuscript, and in the decision to submit the article for publication.

## Conflict of Interest

The authors declare that the research was conducted in the absence of any commercial or financial relationships that could be construed as a potential conflict of interest.
