# Supplementary material for: Inhibition of Female and Male Human Detrusor Smooth Muscle Contraction by the Rac Inhibitors EHT1864 and NSC23766
Source: Front Pharmacol. 2020 Apr 7;11:409. doi: 10.3389/fphar.2020.00409 (PMC7154109; doi:10.3389/fphar.2020.00409)
Supplement: Supplementary file 1 [file DataSheet_1.pdf]

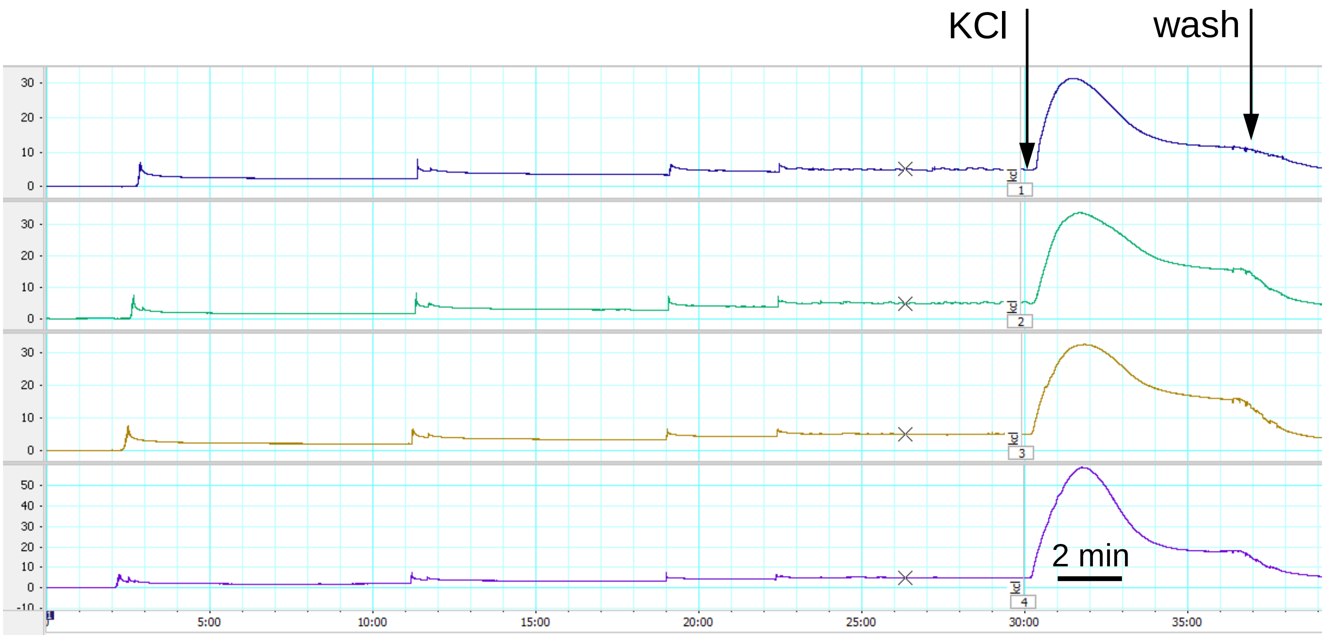

Supplementary figure 1: KCl-induced contractions, original recording from experiment with four samples from the same bladder, showing tonic contractions (peak), and phasic contractions (steady state) followed by washout.

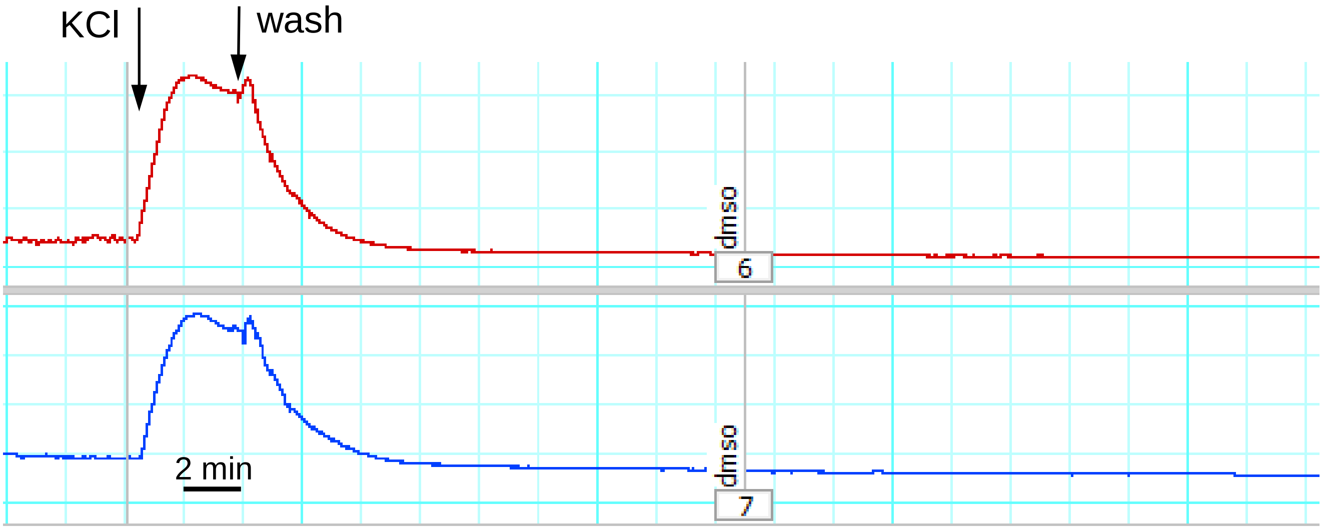

Supplementary figure 2: KCl-induced contractions, showing tonic contractions (peak) and washout starting soon after the peak, followed by stable baselines after washout (original recording of two channels from experiment with four samples from the same bladder) and addition of DMSO (solvent of NSC23766). Stock solutions of NSC23766 had concentrations of 10 mM, so that 100 µl of stock solutions or of corresponding solvent were added to organ bath chambers to attain the final inhibitor concentrations of 100 µM. Each chamber contained 10 ml Krebs-Henseleit solution, resulting in concentrations of 0.99 % for DMSO and for solvent-related water.

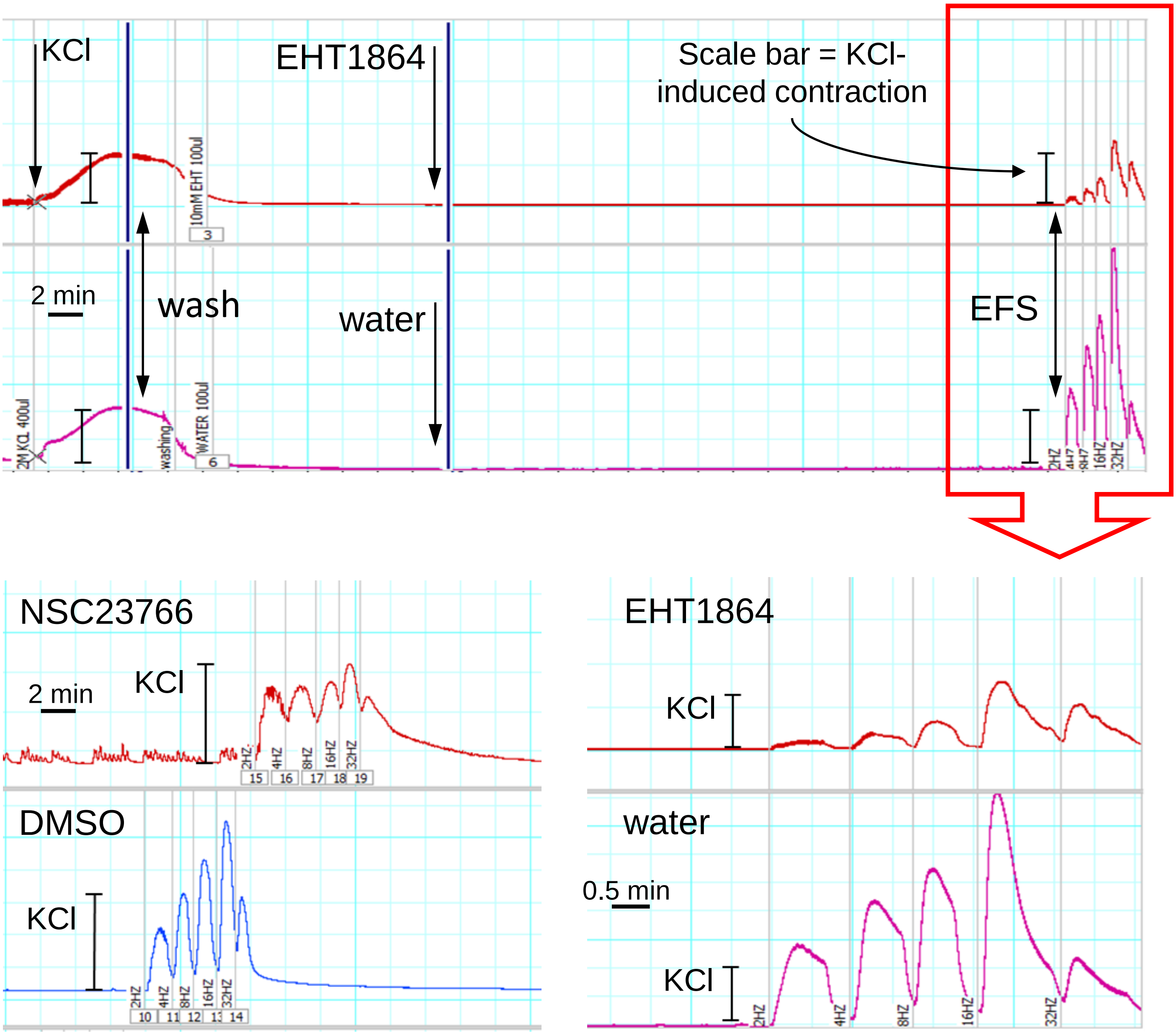

Supplementary figure 3: Effects of Rac inhibitors on EFS-induced contractions. **Upper panel:** Representative experiment (two from four channels), complete from KCl-induced contraction to EFS-induced contractions, addressing effects of EHT1864 and solvent (water) on EFS-induced contraction in samples of a male bladder. **Lower panels:** EFS-induced contractions after application of NSC23766 in a female tissue (left), EHT1864 in a male tissue (right, cutout from upper panel), and corresponding solvents (water for EHT1864, DMSO for NSC23766). Stock solutions of both inhibitors had concentrations of 10 mM, so that 100 µl of stock solutions or of corresponding solvent were added to organ bath chambersto attain the final inhibitor concentrations of 100 µM. Each chamber contained 10 ml Krebs-Henseleit solution, resulting in concentrations of 0.99 % for DMSO and for solvent-related water. Verticale scale-bars = corresponding magnitude of KCl-induced contraction, assessed as shown in supplementary figures 1 and 2, separately for each channel. Each panel shows two from four channels from the same experiment.

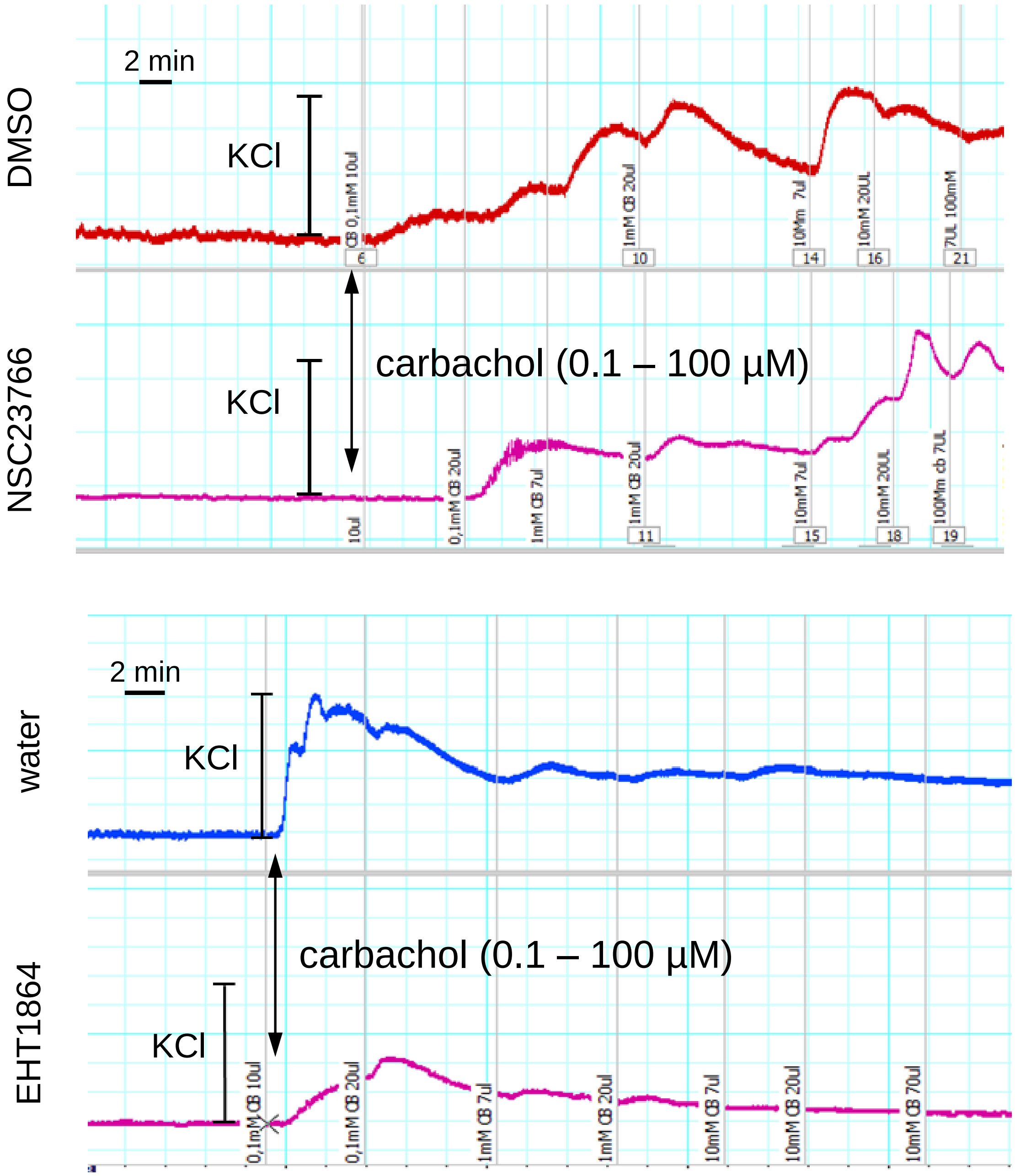

Supplementary figure 4: Effects of Rac inhibitors on carbachol-induced contractions. Shown are concentration response curves for carbachol, after addition of NSC23766 **(upper panel)**, EHT1864 **(lower panel)**, and solvent (both panels; DMSO for NSC23766, water for EHT1864) in male tissues. Stock solutions of both inhibitors had concentrations of 10 mM, so that 100 µl of stock solutions or of corresponding solvent were added to organ bath chambersto attain the final inhibitor concentrations of 100 µM. Each chamber contained 10 ml Krebs-Henseleit solution, resulting in concentrations of 0.99 % for DMSO and for solvent-related water. Verticale scale-bars = corresponding magnitude of KCl-induced contraction, assessed as shown in supplementary figures 1 and 2, separately for each channel. Shown are two from four channels from the same experiment in each panel.
